# Hypertension-Related Gene Polymorphisms of G-Protein-Coupled Receptor Kinase 4 Are Associated with NT-proBNP Concentration in Normotensive Healthy Adults

**DOI:** 10.1155/2012/806810

**Published:** 2012-02-21

**Authors:** Junichi Yatabe, Midori S. Yatabe, Minoru Yoneda, Robin A. Felder, Pedro A. Jose, Hironobu Sanada

**Affiliations:** ^1^Department of Nephrology, Hypertension, Diabetology, Endocrinology and Metabolism, Fukushima Medical University, School of Medicine, 1 Hikarigaoka Fukushima, Fukushima 960-1295, Japan; ^2^Division of Health Science Research, Fukushima Welfare Federation of Agricultural Cooperatives, Fukushima, Japan; ^3^Department of Pharmacology, Fukushima Medical University School of Medicine Fukushima, Japan; ^4^Department of Pathology, University of Virginia Health System, Charlottesville, VA, USA; ^5^Center for Molecular Physiology Research, Children's National Medical Center and George Washington University, School of Medicine & Health Sciences, Washington, DC, USA

## Abstract

G protein-coupled receptor kinase 4 (GRK4) with activating polymorphisms desensitize the natriuric renal tubular D1 dopamine receptor, and these GRK4 polymorphisms are strongly associated with salt sensitivity and hypertension. Meanwhile, N-terminal pro-B-type natriuretic peptide (NT-proBNP) may be useful in detecting slight volume expansion. However, relations between hypertension-related gene polymorphisms including GRK4 and cardiovascular indices such as NT-proBNP are not clear, especially in healthy subjects. Therefore, various hypertension-related polymorphisms and cardiovascular indices were analyzed in 97 normotensive, healthy Japanese adults. NT-proBNP levels were significantly higher in subjects with two or more GRK4 polymorphic alleles. Other hypertension-related gene polymorphisms, such as those of renin-angiotensin-aldosterone system genes, did not correlate with NT-proBNP. There was no significant association between any of the hypertension-related gene polymorphisms and central systolic blood pressure, cardioankle vascular index, augmentation index, plasma aldosterone concentration, or an oxidative stress marker, urinary 8-OHdG. Normotensive individuals with GRK4 polymorphisms show increased serum NT-proBNP concentration and may be at a greater risk of developing hypertension and cardiovascular disease.

## 1. Introduction

Renal tubular dopaminergic system inhibits sodium reabsorption from the filtrate and induces sodium excretion [1]. The dopaminergic action in the kidney is impaired in essential hypertension [2] due to an overactivity of G-protein-coupled receptor kinase type 4 (GRK4), an enzyme that desensitizes dopamine D1 receptor (D1R) [1, 3]. Over-activity of GRK4 is shown to be caused by three activating variants of GRK4, namely, R65L, A142V, and A486V [2, 4], in humans, and we have previously reported that these GRK4 gene variants are associated with salt-sensitive and low-renin hypertension [4].

 In addition to GRK4, gene polymorphisms related to the renin-angiotensin-aldosterone system (RAAS), such as angiotensin converting enzyme (ACE), angiotensinogen (AGT), angiotensin II type 1 receptor (AGTR1), and aldosterone synthase (CYP11B2) [5], have been reported to be associated with hypertension.

 Many studies attempt to link a certain genotype with hypertensive phenotype by establishing a difference in genotype frequency between hypertensive and normotensive patients. However, hypertension is a complex trait that develops slowly and latently, being modulated by many overlapping and counteracting systems. Hence, it is difficult to convincingly establish the causality of gene polymorphisms in hypertension, unless such gene variant is shown to increase blood pressure in transgenic animal models [2]. Given this limitation, it may be beneficial to explore associations between factors postulated to underlie hypertension, such as fluid retention, RAAS overactivation or oxidative stress, and hypertension-related genotypes in those who have not yet developed hypertension.

 N-terminal pro-B-type natriuretic peptide (NT-proBNP) is a peptide made by cardiomyocytes during the formation of brain natriuretic peptide (BNP). NT-proBNP is often used as a biomarker of cardiac volume and pressure load [6–8]. Because NT-proBNP as a marker is very sensitive and dynamic, NT-proBNP may reflect mild fluid retention caused by hypertension-related gene variants.

 Another prohypertensive condition postulated to be associated with many of the gene variants is the over-activation of the RAAS. The increased activity of the RAAS may be due to the increased aldosterone production in some instances [9, 10] and an increase in oxidative stress [11] in others. Therefore, urinary 8-hydroxy-2′-deoxyguanosine (8-OHdG), a marker of oxidative stress, and plasma aldosterone concentration were also measured in this study.

The present study tested the correlations of hypertension-related gene polymorphisms including GRK4 with physiological and chemical indices related to hypertension and atherosclerosis in healthy, normotensive adults.

## 2. Methods

### 2.1. Study Subjects

All protocols were carried out with the approval of Fukushima Medical University Institutional Review Board. Study participants were recruited Japanese volunteers with normal blood pressure (<140/90 mmHg at office or <135/85 mmHg at home) as defined by the Japanese Society of Hypertension Committee for Guidelines for the Management of Hypertension [12] who were working in a hospital in Fukushima Prefecture. Those with cardiovascular disease (subjects receiving regular followup), chronic kidney disease (estimated glomerular filtration rate <60 mL/min/1.73 m^2^ and/or macroproteinuria), or diabetes (HbA1c-JDS < 6.5% and/or receiving medical treatment) were excluded from the study. After providing written consent, study subjects underwent physical examinations and laboratory tests on urine and blood. A total of 97 subjects were enrolled into the study.

### 2.2. Physiological Testing

Cardioankle vascular index (CAVI) and ankle-brachial index (ABI) were measured by VS-1500 N (Fukuda Denshi Co., Ltd. Tokyo, Japan). Augmentation index (AI) and central systolic blood pressure (cSBP) were measured by HEM-9000AI (OMRON Corp. Kyoto, Japan).

### 2.3. Urine and Blood Analyses

Second voided urine after wakeup was collected for analysis. Urinary electrolytes were measured to estimate the amount of daily sodium intake based on the Kawasaki's formula [13, 14]. Urinary albumin and 8-OHdG was measured by latex nephelometry and enzyme-linked immunosorbent assay, respectively. Patient samples for blood analyses were obtained at 8:30 AM after the patients had rested in the recumbent position for 30 minutes. Routine laboratory analyses were performed in the hospital laboratory with an automated method using Hitachi Autoanalyzer 7070 (Hitachi High-Technologies Corporation, Tokyo, Japan). Complete blood count was performed using XT-1800i (Sysmex, Kobe, Japan). Plasma aldosterone concentration was measured by radioimmunoassay. Serum NT-proBNP concentration was measured by electrochemiluminescence immunoassay. Urinary albumin and 8-OHdG, plasma aldosterone concentration, and NT-proBNP were measured by SRL Inc. (Tokyo, Japan).

### 2.4. Genotyping

Variants of GRK4 (R65L A142V, and A486V), AGT M235T, AT1R A1166C, CYP11B2 C-344T, and PAI-1 4/5G were detected by fluorescence probe melting curves [4]. AGT A-6G, ACE I/D were genotyped as previously reported. Detailed protocols have been published elsewhere [15–18].

### 2.5. Statistics

Data are given as Mean ± SD. Correlations between physiological indices and number of GRK4 single nucleotide polymorphisms (SNPs) are assessed by one-way factorial ANOVA. Association between systolic blood pressure and NT-ProBNP levels were analyzed by pearson product-moment correlation coefficient test. Differences in laboratory tests among groups in Table 2 were analyzed by Cochran-Cox test and one-way factorial ANOVA.

## 3. Results

### 3.1. General Characteristics

 Of the 97 subjects enrolled, 74% was female. The mean systolic and diastolic blood pressures at the office were 124.4 ± 16.2 and 79.1 ± 12.4 mmHg, respectively. Since subjects with diabetes were excluded from the study, the average levels of FBS and HbA1c were normal (Table 1).

### 3.2. Association between Physiological Indices and the Number of GRK4 Polymorphisms

**Figure 1:**
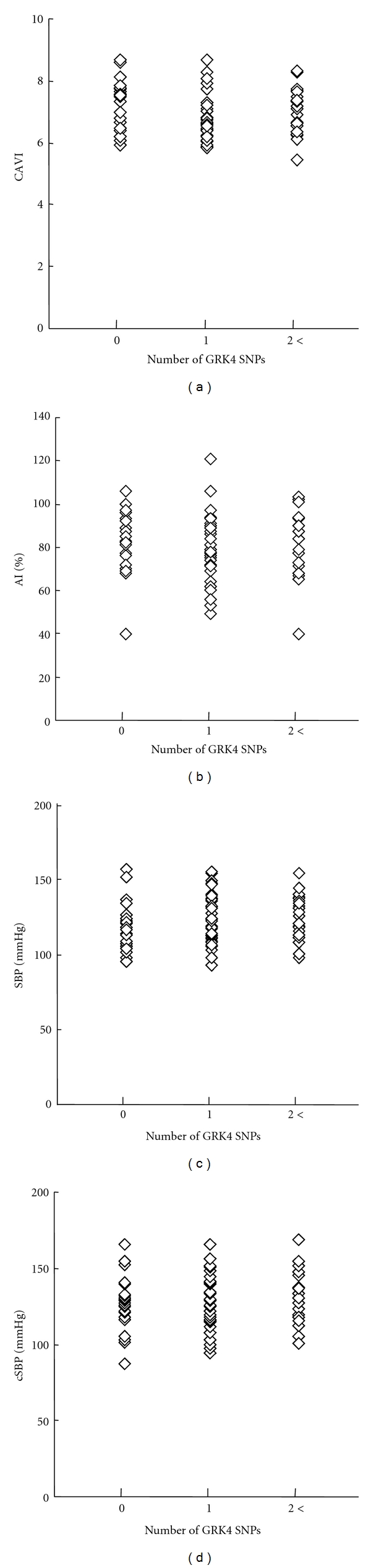
The relationships between number of GRK4 polymorphic alleles GRK4 (R65L, A142V, A486V—LL, VV, VV = 6 alleles) and (a) cardio-ankle vascular index (CAVI), (b) augmentation index (AI), (c) systolic blood pressure (SBP), and (d) central systolic blood pressure.

 CAVI, AI, SBP, and cSBP were measured as physiological indices of atherosclerotic changes. No significant relationships were observed between the number of GRK4 polymorphisms and CAVI, AI, SBP, or cSBP (Figure 1).

### 3.3. Association between NT-proBNP Levels and Number of GRK4 Polymorphisms

**Figure 2:**
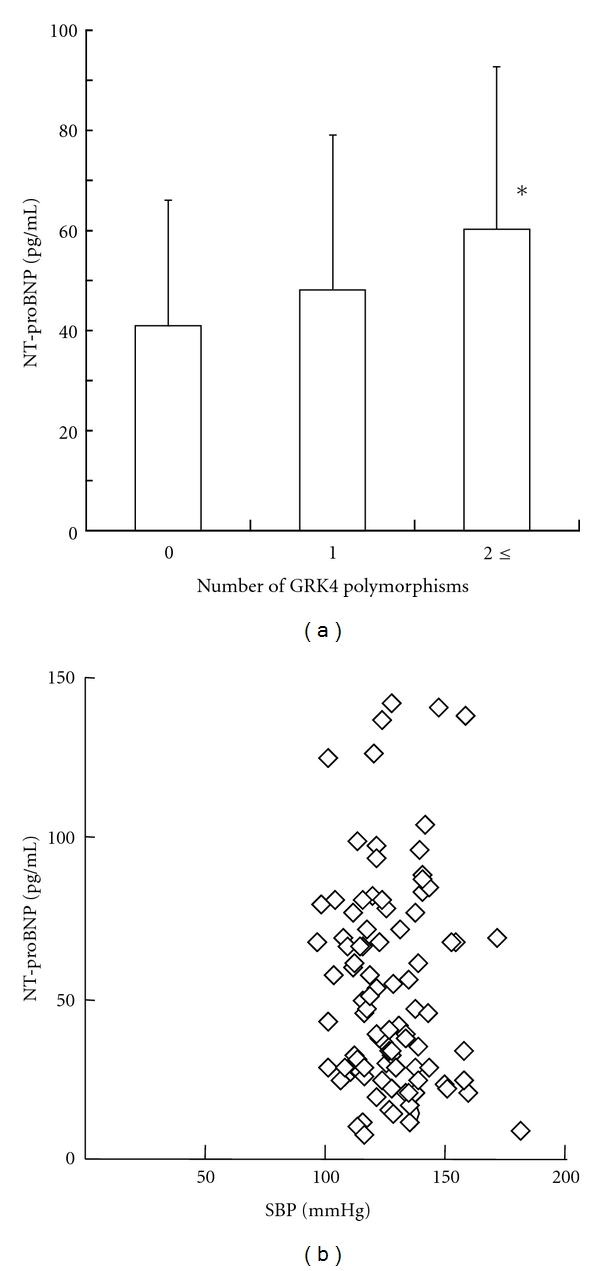
(a) Relationship between the number of GRK4 polymorphic alleles GRK4 (R65L, A142V, A486V—LL, VV, VV = 6 alleles) and NT-proBNP concentration. Number of subjects is indicated in each bar. Subjects with incomplete GRK4 genotyping were not included in the analysis. *P < 0.05 versus GRK4 SNP-free subjects. (b) The relationship between systolic blood pressure (Systolic BP) and NT-proBNP concentration.

 In the subjects who have two or more GRK4 polymorphic alleles, NT-proBNP levels were significantly higher than those with one or no GRK4 polymorphic allele (41.0 ± 5.0 versus 60.2 ± 6.7 pg/mL, *P* < 0.05, Figure 2(a)). This was not due to higher blood pressure, as there was no association between NT-proBNP and office SBP in the healthy normotensives in this study (Figure 2(b)).

### 3.4. Association between the Levels of NT-proBNP, Aldosterone, 8-OHdG, and Hypertension-Related Genotypes Other Than GRK4

 Other hypertension-related genotypes, ACE, AGT, AGTR1, and CYP11B2, were not significantly associated with NT-proBNP concentration (Table 2). Also, none of the hypertension-related polymorphisms examined showed significant association with plasma aldosterone concentration or urinary 8-OHdG, a marker of oxidative stress (Table 2).

## 4. Discussion

 The present study showed for the first time that a significant increase in serum NT-proBNP concentration exists in normotensive, healthy subjects harboring hypertension-related polymorphisms of GRK4, while there was no association between any of the hypertension-related genotypes studied and physiological indices (CAVI, AI, SBP, and cSBP), plasma aldosterone concentration, or an oxidative stress marker, urinary 8-OHdG.

We have previously reported the association between GRK4 polymorphisms (R65L, A142V, and A486V) and salt sensitivity and/or hypertension [19]. In Japanese with untreated, newly diagnosed hypertension, a genetic model based on GRK4 R65L, GRK4 A142V, and GRK4 A486V was 94.4% predictive of salt-sensitive hypertension [4]. Also, in normotensive, young American twins, individuals who were homozygous for haplotype 65L-142V-A486 showed a 1.05 mmHg steeper increase in SBP per year increase in age compared with those homozygous for the most common R65-A142-A486 haplotype [20]. The present study did not find a blood pressure difference in normotensive, Japanese adults possibly because of lack of power due to a smaller sample size. However, the fact that there was a significant increase in serum NT-proBNP concentration among those with GRK4 polymorphisms suggests that salt-sensitive phenotype may be apparent before blood pressure elevation.

Salt sensitivity is associated with an increased morbidity and mortality due to cardiovascular disease even in normotensive population [21]. In patients with stable coronary disease, NT-pro-BNP is a better marker of long-term mortality than conventional cardiovascular risk factors or the degree of left ventricular systolic dysfunction [22, 23]. There are also reports that NT-proBNP is associated with unfavorable cardiovascular outcome in patients with left ventricular hypertrophy [24], geriatric population [25, 26] or diabetes [27]. To determine if GRK4 polymorphisms and NT-proBNP can predict future cardiac risks, prospective studies with larger sample size may be useful.

 Physiological indices, CAVI and AI, are both known to increase with greater number of cardiovascular risk factors and complications [28–30]. AI may be more sensitive than CAVI in detecting small differences in cardiovascular risk factors [31–33]. Also, estimated central systolic blood pressure (cSBP) has shown to be better than brachial blood pressure in assessing atherosclerosis and predicting cardiovascular outcome [34]. However, in this relatively young, normotensive study population, none of the hypertension-related genotypes correlated with cSBP, CAVI, or AI. The lack of association in this study may suggest that detectable atherosclerotic damage or hemodynamic alteration is not one of the early changes during the development of hypertension.

 The major finding of this study is that NT-proBNP concentration was slightly but significantly elevated in the normotensive subjects with more hypertension-related GRK4 polymorphisms. The values of NT-proBNP observed in the present study fall within the normal range, but there have been studies that show slight but significant changes with different conditions. Heringlake et al. examined the effects of saline infusion on healthy men and found a significant elevation of plasma NT-proBNP from approximately 3 pmol/L (25 pg/mL) to 7 pmol/L (59 pg/mL) 8 hours after the infusion [35]. Even though the difference in the values of NT-proBNP is relatively small, the diagnostic benefit may still be apparent.

 It is notable that among the various hypertension-related gene polymorphisms studied, only GRK4 showed a significant correlation with serum NT-proBNP concentration. This finding is in accordance with the proposed mechanism of association between GRK4 and hypertension, as polymorphic GRK4 has been shown to increase D1R phosphorylation, decrease cAMP response to dopamine in renal tubular cells, and impair natriuresis [2, 4], which would lead to sodium and fluid retention.

Another finding from this study is that none of the hypertension-related gene polymorphisms were associated with plasma aldosterone concentration or urinary 8-OHdG. An increase in aldosterone production is postulated to be a mechanistic link between some RAS-related polymorphisms and hypertension [10, 36], and oxidative stress is considered as an important factor in hypertension involving over-active RAAS [37]. However, at least in this study population, no significant difference could be detected in plasma aldosterone concentration or urinary 8-OHdG by different genotypes. An important limitation of this study is that the majority (74%) of study subjects were female, reflecting the high female ratio of hospital workers in Japan. As such, some of the findings may not be directly applicable to the general population.

In conclusion, GRK4 polymorphisms have a close relationship with serum NT-proBNP concentration, which may reflect fluid retention and increased cardiac load. GRK4 may be a potential target to screen for the risk of future hypertension and cardiovascular disease so that lifestyle modifications such as salt restriction can be chosen by at-risk subjects to prevent future adverse events.

##  Conflict of Interests

R. A. Felder and P. A. Jose were awarded a US Patent (no. 6,660,474) on “GRK mutants in essential hypertension” and formed the company Hypogen, Inc.

## Figures and Tables

**Table 1:** Basic characteristics of the subjects.

	Units	Mean	SD
Female ratio	%	74	
Age	years	44.5	9.8
SBP	mmHg	124.4	16.2
DBP	mmHg	79.1	12.4
eGFR	mL/min./1.73 m^2^	89.3	14.0
HDL-C	mg/dL	57.0	14.4
LDL-C	mg/dL	106.5	28.3
FBS	mg/dL	96.5	10.6
HbA1c	%	5.39	0.32
Serum uric acid	mg/dL	4.6	1.3

SBP: systolic blood pressure, DBP: diastolic blood pressure, eGFR: estimated glomerular filtration rate, FBS: fasting blood sugar. HbA1c is represented as the National Glycohemoglobin Standardization Program value.

**Table 2:** Hypertension-related gene polymorphisms and serum NT-proBNP, plasma aldosterone, and urinary 8-OHdG concentration.

	NT-proBNP (pg/mL)		
ACE	II	DI	DD
	46.3 ± 4.0 (42)	57.1 ± 5.8 (43)	46.0 ± 9.6 (12)

AGT A-6G	AA	AG/GG	
	50.7 ± 4.5 (61)	49.2 ± 5.1 (32)	

AGT M236T	MM/MT		TT
	48.1 ± 5.2 (29)		47.8 ± 4.6 (52)

AGTR1 A1166C	AA	AC/CC	
	48.4 ± 3.4 (75)	45.4 ± 17.8 (7)	

CYP11B2 C-344T	CC	CT	TT
	48.7 ± 4.9 (46)	51.7 ± 5.1 (39)	56.6 ± 13.5 (9)

PAI-1	5G5G	4G5G	4G4G
	35.5 ± 4.5 (11)	57.2 ± 5.0 (44)	49.0 ± 5.5 (40)

	Aldosterone (pg/mL)		

GRK4	0	1	2≦
	91.1 ± 7.4 (26)	80.2 ± 5.4 (40)	88.4 ± 8.0 (23)

ACE	II	DI	DD
	91.6 ± 6.3 (41)	75.7 ± 4.5 (43)	89.9 ± 10.4 (11)

AGT A-6G	AA	AG/GG	
	77.8 ± 3.9 (61)	92.2 ± 7.3 (32)	

AGT M236T	MM/MT		TT
	77.5 ± 5.5 (29)		84.5 ± 5.1 (52)

AGTR1 A1166C	AA	AC/CC	
	82.5 ± 4.1 (75)	78.2 ± 9.6 (7)	

CYP11B2 C-344T	CC	CT	TT
	76.7 ± 4.6 (46)	88.1 ± 5.9 (39)	92.9 ± 12.9 (9)

PAI-1	5G5G	4G5G	4G4G
	86.9 ± 11.2 (11)	85.8 ± 5.8 (44)	78.3 ± 4.6 (40)

	8-OHdG (ng/mL/g/crea)		

GRK4	0	1	2≦
	107.5 ± 5.0 (23)	95.8 ± 4.2 (39)	101.0 ± 5.0 (23)

ACE	II	DI	DD
	106.7 ± 4.2 (38)	98.7 ± 3.5 (43)	91.2 ± 7.7 (12)

AGT A-6G	AA	AG/GG	
	97.6 ± 2.9 (59)	105.3 ± 5.2 (31)	

AGT M236T	MM/MT		TT
	103.7 ± 5.5 (28)		98.3 ± 3.1 (50)

AGTR1 A1166C	AA	AC/CC	
	100.7 ± 3.0 (72)	93.9 ± 5.8 (7)	

CYP11B2 C-344T	CC	CT	TT
	102.7 ± 3.7 (44)	100.1 ± 4.4 (39)	91.5 ± 4.5 (8)

PAI-1	5G5G	4G5G	4G4G
	88.1 ± 8.5 (9)	102.2 ± 3.9 (43)	102.2 ± 3.9 (39)

Number of subjects in each group is indicated in parenthesis below the genotype. GRK4: G-protein coupled receptor kinase 4, ACE: Angiotensin converting enzyme, AGT: angiotensinogen, AGTR1: Angiotensin II type 1 receptor, CYP11B2: aldosterone synthase, PAI-1: plasminogen activator inhibitor-1, 8-OHdG: 8-hydroxy-2′-deoxyguanosine. Data are represented by mean ± SE. Cochran-cox test was used for the paired comparison and one-way factorial ANOVA was applied for multiple comparisons.
